# Development of an *in vivo* murine model of perineural invasion and spread of cutaneous squamous cell carcinoma of the head and neck

**DOI:** 10.3389/fonc.2023.1231104

**Published:** 2023-09-07

**Authors:** Priscila O. de Lima, Natasa Broit, Johnson D. Huang, Jae H. Lim, Damien J. Gardiner, Ian S. Brown, Benedict J. Panizza, Glen M. Boyle, Fiona Simpson

**Affiliations:** ^1^ Frazer Institute, University of Queensland, Brisbane, QLD, Australia; ^2^ Cancer Drug Mechanisms Group, Queensland Institute of Medical Research (QIMR) Berghofer Medical Research Institute, Brisbane, QLD, Australia; ^3^ Queensland Skull Base Unit and Department of Otolaryngology, Head and Neck Surgery, Princess Alexandra Hospital, Brisbane, QLD, Australia; ^4^ Envoi Pathology, Brisbane, QLD, Australia; ^5^ Faculty of Medicine, University of Queensland, Brisbane, QLD, Australia; ^6^ Department of Otolaryngology-Head and Neck Surgery, Kaiser Moanalua Medical Center, Honolulu, HI, United States; ^7^ Faculty of Health, Queensland University of Technology, Brisbane, QLD, Australia

**Keywords:** perineural invasion, perineural spread, cutaneous SCC of the head and neck, *in vivo* mouse models, LOXL2

## Abstract

**Introduction:**

Cutaneous squamous cell carcinoma of the head and neck (cSCCHN) can metastasize by invading nerves and spread toward the central nervous system. This metastatic process is called perineural invasion (PNI) and spread (PNS). An *in vivo* sciatic nerve mouse model is used for cSCCHN PNI/PNS. Here we describe a complementary whisker pad model which allows for molecular studies investigating drivers of PNI/PNS in the head and neck environment.

**Methods:**

A431 cells were injected into the whisker pads of BALB/c *Foxn1^nu^* and NSG-A2 mice. Tumor progression was monitored by bioluminescence imaging and primary tumor resection was performed. PNI was detected by H&E and IHC. Tumor growth and PNI were assessed with inducible ablation of LOXL2.

**Results:**

The rate of PNI development in mice was 10%-28.6%. Tumors exhibited PNI/PNS reminiscent of the morphology seen in the human disease. Our model’s utility was demonstrated with inducible ablation of LOXL2 reducing primary tumor growth and PNI.

**Discussion:**

This model consists in a feasible way to test molecular characteristics and potential therapies, offers to close a gap in the described *in vivo* methods for PNI/PNS of cSCCHN and has uses in concert with the established sciatic nerve model.

## 1 Introduction

Cutaneous squamous cell carcinoma represents approximately 20% of all keratinocyte cancers, with about 55% of lesions occurring on the head and neck (1). This is predominantly due to life-long exposure of this area to the sun and damaging effects of ultra-violet radiation. Most cases of cutaneous squamous cell carcinomas of the head and neck (cSCCHN) can be treated with surgery and/or radiotherapy with curative outcomes, unless high-risk features are observed such as perineural invasion (2).

Perineural Invasion (PNI), defined as the invasion of tumor cells into the perineural space of a peripheral nerve (3), predicts poor prognosis in several cancers including pancreatic (4), gastric (5), colorectal (6, 7) and cSCCHN (8–12). Incidental PNI is usually asymptomatic, involves small nerves and is detected on histopathology. Its incidence in cSCCHN ranges from 2%-14% (13–18) and its detection denotes an aggressive tumor with higher rates of local recurrence and lymph node metastases (19–22). PNI cases are usually managed by surgery and radiotherapy. However, PNI can spread to large central nerves away from the initial primary resulting in clinical and radiological signs known as perineural spread (PNS).

PNS or clinical PNI, carries a worse prognosis than incidental PNI, with reduced 5-year local control rates (35-57%) and 5-year disease-specific survival (51-76%) in comparison with incidental PNI (80-90% and 67-90%, respectively) (8, 23–27). The most common nerves involved in PNS of cSCCHN are sensory and/or motor nerves, particularly the trigeminal (V) and facial (VII) nerves (8, 18, 28, 29). Depending on the nerve involved and the extent of the invasion, symptoms may include pain, formication, paresthesia, palsy all indicating a loss of nerve function (30, 31).

The molecular mechanisms of PNI/PNS are not fully understood. Although molecular studies have focused on patient samples (32), the lack of knowledge can, in part, be attributed to the limited available models that represent the natural progression of this path of spread. Well-described PNI models have been reviewed previously (23, 33, 34). Briefly, *in vitro* models have been used to study the interactions between cancer cells and neurons in extracellular matrix (35–37). However, to mimic the tumor-nerve microenvironment more closely, *in vivo* approaches have been developed, including heterotopic models which have been widely used to investigate PNI. In this strategy, cancer cells are injected into specific nerves such as the sciatic nerve, allowing PNI assessment over time through magnetic resonance imaging and histology (Bakst and Wong, (33)); Deborde et al., 2018 (38). However, this model is unsuitable to study the early stages of PNI, especially in the head and neck region. To overcome this, orthotopic models consisting of the implantation of cancer cells into the target organ/tissue (Bakst and Wong, (33)) have been employed. Despite the several strategies to investigate PNI, there is still a lack of realistic *in vivo* models that accurately reflect this metastatic process in specific cancers.

**Figure 1:**
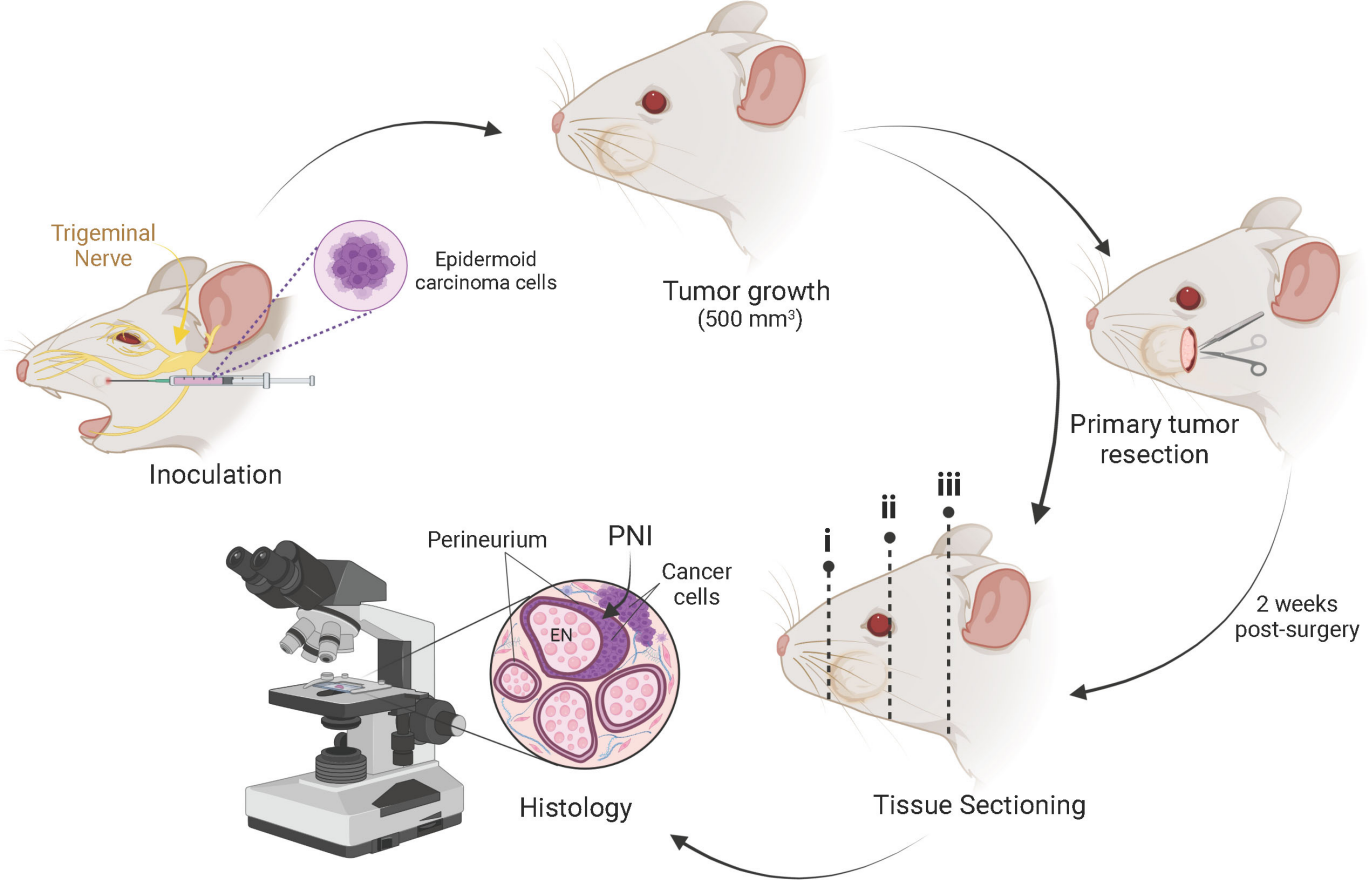
Schematic representation of the mouse model of PNI/PNS of cutaneous head and neck squamous cell carcinoma. Subcutaneous injection of 2 × 10^4^ epidermoid carcinoma cells (A431 or A431-Luc2) was performed into the whisker pad of mice in a 20 µl bolus. Tumors were grown to a maximum volume of 500 mm^3^. Mice were either euthanized for histological assessment of PNI or underwent primary tumor resection. In the latter case, PNI was assessed 2 weeks post-surgery. After decalcification, sectioning of the mouse heads for histological processing was performed in three regions across the head, as indicated by dotted lines i, ii and iii. This method of sectioning was optimized to allow identification of perineural invasion local to the tumor growth, as well as retrograde perineural spread (PNS) along the cranial nerves toward the brain stem. Tissue staining was performed and analyzed by a pathologist to detect PNI/PNS. Image created with BioRender.com.

Here we describe the development of a novel *in vivo* model that mimics the processes of PNI/PNS of cSCCHN (**Figure 1**). Our model allows monitoring of disease progression, testing of potential therapies, besides serving as a useful tool in the study of factors involved in the molecular pathogenesis of PNI. We further demonstrate the utility of our model and compare it to the well-established sciatic nerve model described by Deborde and colleagues (38). We propose that using both models in conjunction may constitute a more appropriate strategy for therapy testing in the context of PNI/PNS of cSCCHN.

## 2 Materials and methods

All the key reagents and resources used in this study can be visualized below in **Supplementary Table 1**.

### 2.1 Cells

Human epidermoid squamous cell carcinoma A431 (ATCC^®^ CRL-1555™, Manassas, VA) cell line was purchased from the American Type Tissue Culture (ATCC). A431 cells were transduced as previously reported (39) to generate A431-Luc2 and A431-Luc2-shLOXL2 cells. Briefly, A431 cells were transduced with pLENTI6/luciferase-expressing lentivirus and selected with puromycin until all control cells were dead. Subsequently, A431-Luc2 cells were transduced with lentivirus containing a tetracycline repressor construct, followed by virus inducibly expressing shLOXL2, as previously described for alternate constructs (Simmons et al., (39)).

Cells were cultured in Nunc™ T75 and T175 cell culture treated flasks with filter caps (Thermo Fisher Scientific, Waltham, MA). A431 and A431- A431-Luc2-shLOXL2 were cultured in RPMI-1640 medium (Life Technologies, GIBCO, Carlsbad, CA) supplemented with 10% (*v*/*v*) heat-inactivated fetal bovine serum (FBS, Life Technologies), 3 mM 4-(2-hydroxyethyl) piperazine-1-ethanesulphonic acid (HEPES), and antibiotics (100 IU/ml penicillin, 100 μg/ml streptomycin) (Life Technologies, GIBCO). A431-Luc2 cells were grown in Dulbecco’s Modified Eagle’s Medium: Nutrient Mixture F12 (DMEM-F12) (Life Technologies, GIBCO) supplemented with 10% (v/v) heat-inactivated FBS, 2 mM of L-Glutamine (Life Technologies, GIBCO) and 10 mM of HEPES (Life Technologies, GIBCO). Cells were maintained under regulated conditions in a humidified incubator at 37°C, 95% humidity and 5% CO_2_. Cells were sub-cultured twice-weekly using 0.25% trypsin with 3.8% versene in Dulbecco’s PBS or 0.25% trypsin-EDTA at 37°C for approximately 5 min. Mycoplasma tests were performed monthly using PCR or MycoAlert mycoplasma detection kit (Lonza, Basel, Switzerland) and were always negative. Cell line identity was verified by Short Tandom Repeat (STR) profiling with the GenePrint^®^ 10 System (Promega, Madison, WI) according to the manufacturer’s instructions.

### 2.2 Mice

All experimental mouse work was performed in agreement with the Australian code for the care and use of animals for scientific purposes (the Code) and the National Health and Medical Research Council (NHMRC). Animal procedures were approved by the QIMR Berghofer Animal Ethics Committee (Project number: P343, QIMR Berghofer AEC approval number: A0106-040M) and by the University of Queensland Animal Ethics Committee (UQ Health Sciences AEC approval number: UQDI/456/17). As there are no gender related considerations, male and female animals were used throughout the study. Five-week-old male BALB/c *Foxn1* ^nu^ mice (*A*/*A Tyrp1b*/*Tyrp1b Tyrc*/*Tyrc*, MHC haplotype (H2K^d^) and C5 normal; Product code: BCNU; RRID : MGI:5652590) were purchased from the Animal Resources Centre (Murdoch, Western Australia) and were housed at QIMR Berghofer Medical Research Institute animal facility. Six to ten-week-old male and female NSG-A2 mice (NOD.Cg-Mcph1Tg (HLA-A2.1)1Enge Prkdcscid Il2rgtm1Wjl/SzJ; Stock#009617; RRID : IMSR_JAX:009617) were obtained from the Radford laboratory (Mater Medical Research, Brisbane, QLD, Australia), originally sourced from Jackson Laboratory (Bar Harbor, ME). The animals were housed in the Translational Research Institute (TRI) animal facility. All mice were maintained under specific pathogen-free conditions on a 12-hour light/dark cycle, with freely available food and chlorinated water. Health status was monitored daily.

### 2.3 Western blotting

Western blotting was performed as described previously (40). Antibodies used in this study were: rabbit anti-human LOXL2 [EPR12733] (Abcam, Cambridge, UK; 1:1000 dilution) and anti-β-actin (Cell Signaling Technologies (CST), Denvers, MA; 1:2000 dilution). Anti-rabbit secondary (Cell Signaling Technologies; 1:1000 dilution) was for detection.

### 2.4 *In vitro* bioluminescence assay

Luminescence of A431-Luc2 cells (passage 7 and 11) was assessed prior to tumor challenge. Cells were seeded in duplicate in a black 96-well plate by 2-fold serial dilution starting with 40,000 cells. Cells were allowed to attach for 4 hours. A stock of D-luciferin potassium salt (Gold Biotechnology, Olivette, MO) 15 mg/ml was initially prepared, 200 µl of luciferin stock (15 mg/mL) were added to 10 ml of growth media, then 100 µl of this mixture were added to each well for a final concentration of 150 µg/ml. Luminescence was quantified as total flux (photons/second) using the IVIS spectrum optical imaging system and the Living Image^®^ software 4.5.5 (Perkin Elmer, MA, USA). Data were analyzed using GraphPad Prism software v7.03 (Boston, MA).

### 2.5 Preparation of cell lines or mouse inoculation

Once A431, A431-Luc2, and A431-Luc2-shLOXL2 cell cultures reached about 80% confluency, cells were detached from the culture vessel as described above and cell number was determined using a hemocytometer. Cells were centrifuged at 1,500 rpm for 5 min at 4°C. The resulting cell pellet was resuspended in either 1× PBS or RPMI-1640 with 10% FBS to achieve the desired cell number. Cells were prepared immediately prior to their need and were stored on ice up until point of injection.

### 2.6 Subcutaneous inoculation of mice with A-431, A-431-Luc2 and A431-Luc2-shLOXL2 cells

Experimental procedures were performed in a laminar flow hood using aseptic technique in accordance with institutional standing operating protocols. Prior to injection, mice were anesthetized with 4-5% isoflurane and O_2_ flow rate of 0.5 L/min. Once unconscious, animals were maintained in 2% isoflurane and O_2_ flow rate of 0.5 L/min. Using a Terumo^®^ U-100 insulin syringe (31G x ½” needle), the cell suspension was delivered in a 20 µl bolus subcutaneously and unilaterally into the whisker pad of each mouse (**Figure 1**). Injections were performed with the needle aiming toward the snout, parallel to the face, at an angle less than 30° to avoid injecting cells directly into the V_2_ nerve, infraorbital foramen or through the nasal cavity. The appearance of a small translucent bleb following injection confirmed correct placement of the cells into the subcutaneous junction of the skin overlying the whisker pad. All mice were monitored until they fully recovered from the anesthesia.

### 2.7 Mouse monitoring and tumor measurements

Mice weight and tumor measurements were monitored twice per week and monitoring became more frequent as tumor size increased. Tumor length (mm) and width (mm) were measured using digital calipers. Tumor volume (mm^3^) was calculated using the equation below:


$$Tumor\;volume\;{\left(mm\right)}^{3}=\frac{\left(length\;\times \;width^{2}\right)}{2}$$

Tumors were grown to a volume of 500 mm^3^, however mice were culled sooner if the animal began indicating signs of distress, behavioral change or if the tumor had become ulcerated.

### 2.8 Primary tumor resection

A431-Luc2 tumor-bearing NSG-A2 mice underwent surgical excision when tumor volume reached 200-500 mm^3^. All the surgeries were performed in a safety cabinet observing aseptic technique. Surgical gown, facemask, and gloves were worn for these procedures. Previously autoclaved surgical instruments as well as other materials were placed inside the safety cabinet and irradiated with UV light. Surgical instruments were wiped clean and disinfected with 70% ethanol for at least 15 minutes between mice (41). Maximum of two sequential surgeries were carried out. Mice were weighed and anesthetized intraperitoneally (25G needle) with 100 mg/kg of ketamine and 10 mg/kg of xylazine (Clifford Hallam Healthcare, Wynnum, QLD, AUS) solution at a bolus volume of 1% of animal’s bodyweight (10 ml/kg). Anesthesia duration ranged from 20-30 min. A quarter of the initial anesthetic dose was given to animals when surgery could not be finished in 30 min (42). To assess the anesthetic depth, withdrawal reflex was observed by carefully pinching the animal’s toes and paws with forceps. Their respiratory frequency was monitored before and during the procedure. A heating lamp was used to maintain the animal’s temperature during surgery. Once loss of consciousness was observed, animals were prepped for surgery. Eye ointment was applied on both eyes to preserve eye moisture. Each mouse was placed on its lateral where the tumor on the whisker pad is facing upwards. The position of the animal was secured with a hypoallergenic tape. Animals were then covered with a sterile clear plastic drape to prevent contamination during surgery.

A small area at the incision site posterior to the whisker pad, was carefully shaved without compromising whiskers. The incision site was disinfected with povidone-iodine antiseptic. Using scissors, an initial small incision (~ 2-3 mm) was made ipsilateral to the tumor and posterior to the whisker pad, perpendicular to the nose, to facilitate a caudo-cranial skin flap. The incision site is indicated in **Figure 1**. Tumor was carefully detached from underlying tissue and overlying skin by blunt dissection using vannas-type micro-scissors and tweezers style 5 (ProSciTech). In the event of bleeding, cotton tips and a cautery pen (Gemini, tip length 5 mm, Able Scientific, Perth, Western Australia) were used to achieve hemostasis. Resected tumor was stored in 1 × PBS on ice for later tissue fixation in 4% paraformaldehyde. The skin incision was closed with Dermabond Advanced^®^ Topical Skin Adhesive surgical glue (Ethicon, Johnson & Johnson, NJ) only or 6-0 vicryl rapide sutures 6-0 (Ethicon) plus Dermabond Advanced^®^. Animals were then placed in a clean cage below a heating light to adjust the body temperature. Duration of immobilization was approximately 45-60 minutes. Buprenorphine 0.05 mg/kg (Clifford Hallam Healthcare) was administered subcutaneously (31G needle) post-surgery and 8-12 hourly for 2 days post-operative. Mice were given two subcutaneous injections of 50 μl of saline after surgery and on the following day to maintain body fluids. Animals were kept in individual cages and placed into a ventilated recovery cabinet overnight. Wounds were carefully cleaned with saline and kept moisturized with Bepanthen^®^ antiseptic cream (Bayer Pharmaceutics, Leverkusen, DEU), to help soothe the damaged skin and prevent infection. Animals were given soft diet (DietGel boost, Clear H_2_O, Westbrook, ME) to facilitate their eating and were monitored twice daily during the post-operative period.

### 2.9 Live imaging using IVIS spectrum optical imaging system

IVIS optical imaging system was used to monitor tumor growth when using luciferase expressing cells. On the first week post-primary tumor resection, mice were injected intraperitoneally with 200 μl of 15 mg/ml D-luciferin potassium salt (Gold Biotechnology, Olivette, MI). Mice were anesthetized with 2.5% isoflurane. Using field of view (FOV) A or B: 4 cm, images were taken at every minute for 5-10 min to observe saturation of luciferase. Luminescence in the region of interest (ROI) was quantified and measured as total flux (photons/second). Living Image^®^ software v4.5.5 (Perkin Elmer, MA) was used. Data were analyzed and graphs plotted using GraphPad Prism software v8.3.1 (Boston, MA).

### 2.10 Tissue histology

Animals were euthanized by CO_2_ asphyxiation, followed by cervical dislocation. The heads were separated from the body by a cut through the cervical spine at the base of the skull. Heads were then immediately transferred to 10% neutral buffered formalin (Australian Biostain, Taralgon, Victoria, AUS) solution for fixation for a period of 24-48 hours at 4°C. The heads were then transferred to a solution of 15% EDTA in 10 mM phosphate buffer for bone decalcification for 3-4 weeks. The progress of decalcification was routinely checked after three weeks by probing the muzzle for residual hard bone. Once the decalcification process was complete, mouse heads were washed under fresh running H_2_O to remove excess EDTA. Heads were then processed into paraffin wax. Three major transverse sections of the paraffin-embedded head were produced (at 90° to the plane of the maxillary division of the trigeminal nerve; see **Figure 1**) and three sections were again embedded into paraffin. This method of sectioning allows for the identification of varying extents of PNI. The sections capturing tissue posterior to the tumor (**Figure 1**, dotted lines i-ii) allow a histopathologist to track perineural spread (PNS; representing clinical perineural invasion) to named nerves and the brain stem. It also allows the identification of PNI local to the primary tumor (**Figure 1**, dotted line iii). Tissue sections were cut at a thickness of 3-4 μm for hematoxylin and eosin and immunohistochemistry staining.

### 2.11 Tissue staining

The hematoxylin and eosin (H&E) stain was performed as per standard laboratory protocol. Tissue sections for immunohistochemistry (IHC) were affixed on positively charged slides. Briefly, IHC sections were air-dried overnight at 37°C. Sections were then de-waxed, rehydrated, and incubated in 2% H_2_O_2_ for 10 min. Antigen retrieval was performed in 10 mM citric acid buffer for 5 min at 125°C (cytokeratin AE1/AE3 only), followed by tissue cooling and wash steps in Tris-buffered saline (TBS). Endogenous peroxidase activity was blocked by incubating sections in a solution of 1% H_2_O_2_ in TBS for 10 min, followed by a series of wash steps, initially in gently running H_2_O and subsequently in TBS 0.05% Tween-20. Sections designated for the S100 stain were blocked in Background Sniper (Biocare Medical, Concord, CA). Tissue sections to be stained with cytokeratin AE1/AE3 were incubated in 10% normal goat serum. Excess blocking buffer was decanted, and sections were washed in TBS 0.05% Tween-20. Primary antibody anti-pan cytokeratin (clone AE1/AE3, Agilent DAKO, Santa Clara), was diluted at 1:300 in Da Vinci Green antibody diluent (Biocare Medical) and applied overnight at RT. This was followed by secondary incubation with MACH2 anti-mouse HRP (Biocare Medical) for 30 min at RT. Primary antibody anti-S100 protein (Novacastra, Leica Biosystems, Wetzlar, DEU) was diluted 1:160 in Da Vinci Green and was applied for 60 min at RT. Secondary antibody was MACH1 Universal anti-rabbit HRP (Biocare Medical) and was applied for 45 min at RT. Sections were then washed in TBS before incubating sections corresponding to both antibodies in 3,3’-diaminobenzidine (DAB) with H_2_O_2_ as substrate for 5 min at RT colour development. Sections were then washed in H_2_O to remove excess chromogen, before being counterstained with Mayer’s hematoxylin, dehydrated, cleared with xylene and finally mounted.

For the dual IHC staining, samples were incubated with citrate pH 6.0 for 15 minutes at 90°C for antigen retrieval. They were blocked with Mouse on Mouse (M.O.M.) Blocking Reagent (Biocare Medical) and Sniper/2% BSA for 10 minutes. Primary antibodies anti-rabbit S100 (Novacastra, Leica Biosystems) and mouse anti-human pan cytokeratin (clone AE1/AE3, Agilent DAKO) were diluted in Da Vinci Green (1:250) and incubated for 1 hour at RT. Secondary incubation was performed with MACH2 anti-rabbit HRP and MACH2 anti-mouse HRP polymer (Biocare Medical) for 30 minutes at RT. S100 was stained with DAB for 5 minutes and 0.5% peroxide for 10 seconds, and pan cytokeratin with Vector Immpact Purple (Abacus ALS, Cannon Hill, QLD, AUS) for 5 min. Slides were scanned on the Aperio ScanScope XT (Leica Biosystems) or VS120 Olympus Slidescanner (Olympus Life Sciences) at 40× magnification. Images were visualized using the Aperio ImageScope (Leica Biosystems) and the Olyvia (Olympus Life Sciences) software.

### 2.12 Establishment of the sciatic nerve model for PNI/PNS of cSCC

A431-Luc2 cells were prepared as described above. Injection of cancer cells into the sciatic nerve of mice was performed as per (38) and followed the standard operating procedures for animal surgical techniques. NSG-A2 mice were anesthetized with 4% isoflurane and maintained under 2.5% isoflurane. Using a dissecting microscope in a safety cabinet and a custom-made Hamilton syringe (10 µl model 701RN, Hamilton, Reno, NV), 50,000 A431-Luc2 cells in 2 μl of PBS were injected distally into the sciatic nerve of mice over 5 s. One mouse was injected with only PBS (control). Animals were given 0.05 mg/kg buprenorphine (Clifford Hallam Healthcare, Wynnum, QLD, AUS) and 5 mg/kg carprofen (Zoetis, Parsippany-Troy Hills, NJ) s.c. after surgery, followed by 0.05 mg/kg buprenorphine at 12 h and 5 mg/kg carprofen at 24 and 48 h post-surgery. In case mice showed any signs of pain or distress, 5 mg/kg carprofen s.c. was administered every 24 h. Mice were monitored daily.

To observe the progression of tumor growth and spread along the sciatic nerve, live imaging using the IVIS machine was carried out on day 7, 12, 21, and 28 post-surgery. Images were taken using field of view (FOV) C: 13 cm. Three animals were euthanized on day 7, one on day 12 and two on day 28, including the control. Both injected and non-injected sciatic nerves were dissected post-euthanasia and frozen in optimal cutting temperature compound. Samples were sectioned (5 μm thickness) longitudinally on the cryostat and stained with H&E. Slides were scanned using the VS120 Olympus Slidescanner (Olympus Life Science) at 40× magnification. The length of invasion was measured using the software Fiji (43). Three sections of each sample were analyzed to obtain the average length of invasion. Graphs were plotted using GraphPad Prism software v8.3.1.

### 2.13 Determination of perineural invasion and spread

Stained slides were examined by a histopathologist (I.S.B.) in a blinded fashion to confirm the presence of PNI/PNS. The cases were described as positive for PNI/PNS, tumor surrounding nerve without PNI and no PNI. PNI was considered positive only in cases where the perineural space was infiltrated by tumor and where the involved nerve was at, and extending beyond, the periphery of the tumor, at the junction of tumor border and nerve bundle of the whisker pad (i.e. PNS) as well as in the tumor bulk.

## 3 Results

### 3.1 Assessing spread of A431-Luc2 cells using the sciatic nerve model of PNI

**Figure 2:**
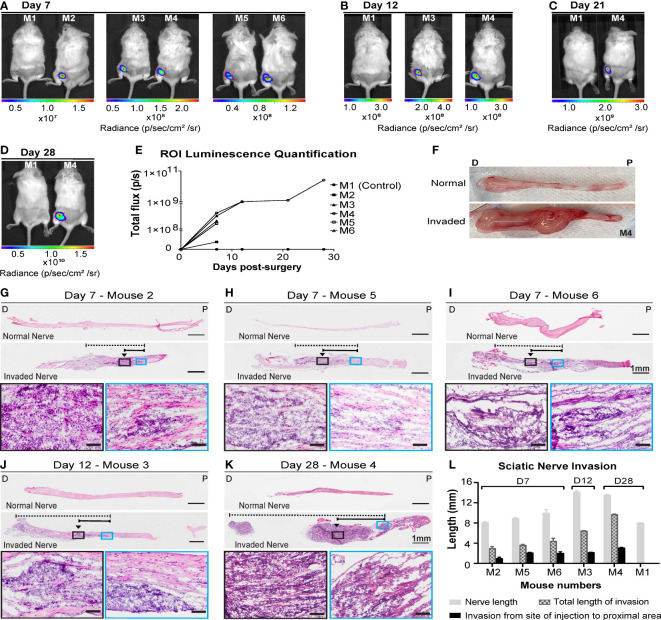
Live imaging and histological analysis of cutaneous SCC cell invasion into the sciatic nerves of NSG-A2 mice. A431-Luc2 cells were injected into the sciatic nerve of 5 NSG-A2 mice. Mouse 1 was injected with PBS only (control). Mice (n=6) were injected via IP with luciferin (15 mg/mL), anesthetized with 2.5% isoflurane, and imaged on days **(A)** 7, **(B)** 12, **(C)** 21 and **(D)** 28 days post-injection with A431-Luc2 cells. Mice were imaged using IVIS spectrum optical imaging system, Living Image^®^ software v4.5.5, field of view: 13 cm. Images show the highest luminescence level representing luciferase saturation. Radiance (p/sec/cm^2^/sr) is represented by the colour scale. **(E)** Region of Interest (ROI) luminescence quantification measured by total flux (photons/second). Data shown as individual values. **(F)** Normal and invaded sciatic nerve of mouse 4 after dissection. P=Proximal; D=Distal. H&E longitudinal sections of sciatic nerves on **(G–I)** 7, **(J)** 12 and **(K)** 28 days post-injection with A431-Luc2 cells. Slides were scanned at 40× magnification using the VS120 Olympus Slidescanner and the software Fiji was used to measure the length (mm) of invasion in the nerve. Black arrows indicate the approximate site of injection. Dashed and solid lines indicate the total length of invasion and the invasion from the site of injection to the proximal spinal cord area, respectively. Scale bars: 1 mm and 100 µm (magnified images in the black and blue boxes). P=Proximal; D=Distal. **(L)** Sciatic nerve invasion measured by length (mm). Data represent the mean ± SEM of 3 sections per sample (n=6). Graphs were plotted using GraphPad Prism v8.3.1. Data shown are the results of a single experiment.

In our adaptation of the sciatic nerve model, cSCC A431-Luc2 cells were injected into the sciatic nerve of NSG-A2 mice. Live imaging confirmed A431-Luc2 cells in the injection site of all animals at day 7 post-surgery, except the control (mouse injected with PBS) (**Figure 2A**). Region of Interest (ROI) luminescence quantification was used to monitor progression of A431-Luc2 tumor growth and spread in the sciatic nerve (**Figures 2A–E**). Luminescence increased with time resulting from tumor growth inside the nerve (**Figure 2E**). Three mice were euthanized on day 7 to assess the degree of invasion. No tumor growth outside the nerve was observed, indicating that the injection was done properly and there was no spill of cells outside the nerve. No infections and no nerve damage were observed, as animals were walking normally. On day 12, mouse 3 was euthanized as it was showing signs of distress and weight lost. No tumor growth was observed outside the nerve. The mouse injected with PBS (control M1) and one mouse injected with A431-Luc2 cells (M4) were kept until 28 days post-surgery. Differences between normal and invaded nerve of animal M4 can be observed macroscopically after dissection. Invaded nerve showed increased thickness in comparison with normal nerve and formation of a round tumor mass on the site of the injection on day 28 (**Figure 2F**). The H&E staining confirmed 100% penetrance of A431-Luc2 cells in the sciatic nerves of challenged animals (**Figures 2J–L**). On day 7, the average of total invasion length and the invasion length from the site of injection to proximal area (spinal column) was 3.7 mm and 1.7 mm, respectively (n=3) (**Figures 2G–I, L**). On day 12, mouse 3 started limping and was euthanized. Mouse 3 presented with approximately 6.5 mm of total nerve invasion length and 2.2 mm of invasion from the site of injection to proximal area (**Figures 2J, L**). On day 28, mouse 4 showed 9.7 mm of total invasion length and 3.1 mm of invasion length from the site of injection to proximal area (**Figures 2K, L**). Histology indicated that total extension of nerve invasion increased over time (**Figure 2L**), corroborating with luminescence quantification data (**Figures 2A–E**).

### 3.2 Optimization of cell number for tumor growth in the whisker pad mouse model

**Figure 3:**
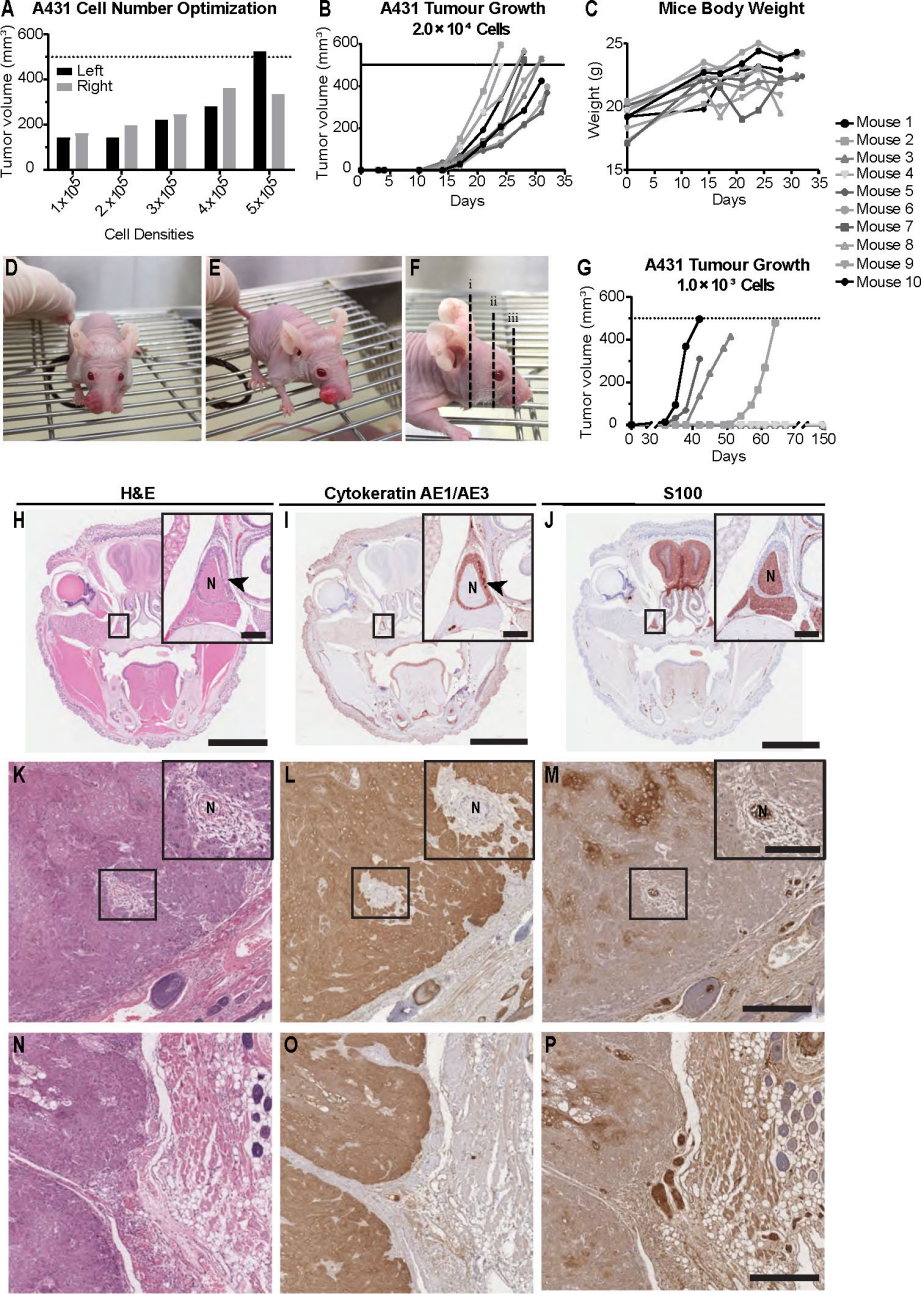
Mouse model of perineural invasion and spread of cutaneous SCCHN using Balb/*c Foxn1^nu^* mice. **(A)** A431 epidermoid carcinoma cell number optimization. Cells were injected bilaterally into the whisker pad of five Balb/*c Foxn1^nu^* mice at varying densities (1.0 × 10^5^, 2.0 × 10^5^, 3.0 × 10^5^, 4.0 × 10^5^ or 5.0 × 10^5^ cells/site). Tumors were allowed to grow for 19 days, after which time mice were culled. No animals in this cohort developed perineural invasion (PNI) or spread (PNS). Data represents individual tumor volumes of left and right whisker pad at day 19. **(B)** A431 tumor growth in mice injected with 2.0 × 10^4^ cells/site. Tumor growth was evident by day 14 and was consistent across the group. All mice (n=10) were deceased by day 32 with the majority exhibiting tumors of approximately 500 mm^3^. **(C)** Mice body weight. Mouse weights (g) of the 2.0 × 10^4^ cell inoculum cohort (n=10) remained unaffected despite increasing tumor burden local to the nasal and oral cavity. Dashed lines represent maximum approved tumor volume (500 mm^3^). **(D, E)** Representative images of tumor growth in the whisker pad of Balb/*c Foxn1^nu^* mice. **(F)** Representative sectioning method for histological assessment of PNI and PNS. **(G)** A431 tumor growth in mice injected with 1.0 × 10^3^ cells/site; 4/10 mice developed tumors. The first tumor was observed 23 days after cell injection and the remaining tumors appeared in the 19 consecutive days (day 35, 42, 51). The remaining six mice were monitored for a period of 150 days from point of injection but failed to form tumors. **(H)** Hematoxylin and eosin (H&E) stain of section with a nerve exhibiting PNI. **(I)** Cytokeratin AE1/AE3 stain showing localized tumor infiltration into the perineural space of the nerve. **(J)** S100 stain showing involved nerve. **(K)** H&E stain of an uninvolved nerve with tumor surrounding the perineural space. **(L)** Cytokeratin AE1/AE3 stain showing tumor surrounding the epineurium with no evidence of tumor infiltration into the perineural space. **(M)** S100 stain of uninvolved nerve. **(N)** H&E showing junction between tumor tissue and underlying whisker pad tissue. **(O)** Cytokeratin AE1/AE3 stain of tumor tissue. **(P)** S100 stain showing nerve bundle at the margin of the tumor with no tumor infiltration into the perineural space. Arrowheads indicate PNI. N: nerve. Scale bars: 3 mm **(H–J)**, 200 µm (**H–J** inserts), 500 µm **(K–P)**, 100 µm (**K–M** inserts).

In order to establish a murine model that reflects PNI/PNS of cSCCHN more closely, we optimized cSCC tumor growth in the whisker pad of mice. We examined cell number inoculum, tumor growth rate and the propensity for metastasis. Initially, we injected 7.0 × 10^5^ cells into the whisker pad of nude mice. Solid tumors formed well in this region; however, tumor growth was rapid and the experiment was terminated 20 days after injection due to the ethical endpoint being reached. No PNI was observed in this group, although nerves were visible adjacent to tumor margins (data not shown). It was hypothesized that rapid tumor growth may not allow the opportunity for PNI to occur, as occurs in patients with cSCCHN. Hence, a smaller initial number of cells was inoculated to allow tumors to form more slowly, and potentially increase the opportunity for invasion into the perineural space. For the subsequent experiment, a range of starting cell densities (1.0 – 5.0 × 10^6^ cells/site) (**Figure 3A**) was injected bilaterally into the whisker pads of five mice to determine the viability of such densities. Again, tumors grew rapidly and all mice were culled by day 19 following initial injection due to ethical considerations. Data presented shows final tumor volumes at the end-point. No PNI or PNS was identified in this cohort. We hypothesized that 2.0 × 10^4^ cells/site may be a potential starting cell number to balance tumor growth with prospective development of PNI.

### 3.3 Perineural invasion and spread observed with moderate cell number inoculum in the whisker pad of BALB/c Foxn1nu mice

Mice injected with 2.0 × 10^4^ A431 cells/site had tumors formed by day 14 post-injection (**Figure 3B**) and mouse measurements indicated steady and consistent tumor growth in all ten mice. All tumors had reached maximum allowable size (500 mm^3^) by day 32. Representative images of tumor growth and tissue sectioning are shown in **Figures 3D–F**. Examples of PNI histology are displayed in **Figures 3H–P**. Examination of stained slides from each mouse found that 1/10 mice (10%) had developed PNI/PNS (arrowheads on **Figures 3H–J**). Several of the cohort additionally had tumor surrounding nerve but no invasion into the perineural space was seen in these cases (**Figures 3K–M**). **Figures 3N–P** represent a negative case, where the nerve bundle is observed at the margin of the tumor with no tumor infiltration into the nerve.

### 3.4 Mouse well-being is not affected during tumor growth in the whisker pad

To determine if tumor growth had any effect on feeding or water intake, mouse weights were measured for the 2.0 × 10^4^ cell inoculum cohort. BALB/c *Foxn1* ^nu^ mice did not show any weight loss throughout the experiment (**Figure 3C**), indicating that mice were able to continue feeding normally. One mouse from the same cohort had to be culled prematurely (final tumor volume 367.8 mm^3^) due to poor clinical signs (i.e. rapid breathing). One mouse developed an ulcerated tumor so was also culled early (final tumor volumes 396.0 mm^3^). Most mice with tumors in this region tolerated the tumor burden well, with little to no impact on their overall well-being as assessed using an approved clinical scoring matrix.

### 3.5 Poor tumor growth observed in the whisker pad of cohort injected with low cell number inoculum

To assess if a low starting cell number would alter the rate of PNI/PNS, ten mice were injected with 1.0 × 10^3^ cells/site (**Figure 3G**). Only 4/10 (40%) mice successfully formed tumors. As expected, the time it took for the tumors to begin growing was longer than the 2.0 × 10^4^ cohort. However, the tumor growth was inconsistent, and the onset of growth was highly staggered. Two tumors were detectable on day 32, one on day 42 and one on day 51. Mice were culled on days 42, 51 and 64. Examination of slides found no evidence of PNI in any of the mice that had developed tumors.

### 3.6 A431-Luc2 tumor growth in the whisker pad of NSG-A2 mice

**Figure 4:**
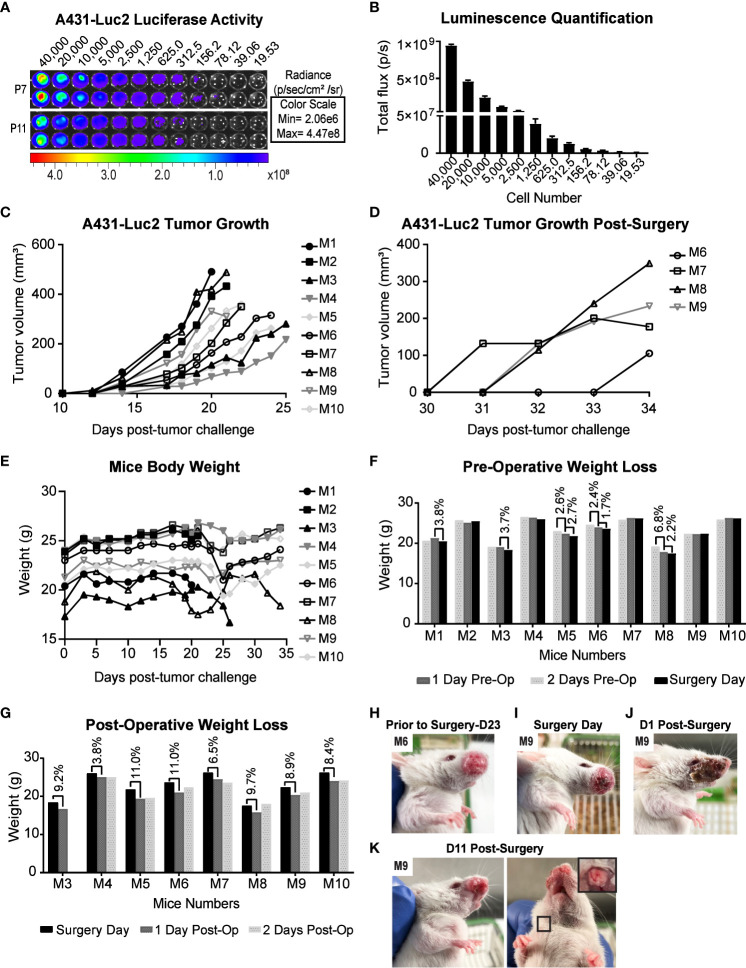
Preclinical model for PNI/PNS of cutaneous SCC of the head and neck using NSG-A2 mice. **(A)** Luciferase activity in A431-Luc cells analyzed by IVIS spectrum optical imaging system. Serial dilution of A431-Luc2 cells (passage 7 and 11) was performed in duplicate. After four hours, luciferin was added to the wells at the final concentration of 150 µg/ml. **(B)** Luminescence Quantification. Luminescence was measured by the total flux (photons/second) and was proportional to the cell number. Data represent mean with SEM. **(C)** A431-Luc2 tumor growth in NSG-A2 mice prior to surgery. Tumors (n=10) were measured three times weekly and resected when they were 200 to 500 mm^3^ (Days 20-25 post-tumor challenge). **(D)** A431-Luc2 tumor growth post-surgery. **(E)** Mice body weight (g). **(F)** Pre-operative weight loss (g). **(G)** Post-operative weight loss (g). Animals exhibited weight loss of 3.8-11% on the first day post-surgery before regaining weight from Day 2 post-surgery. **(H)** A431-Luc2 tumor-bearing mouse prior to surgery (day 23 post-tumor challenge) showing intact skin on the tumor site. **(I)** A431-Luc2 tumor-bearing mouse prior to surgery showing skin redness on the tumor site. Mouse 9 underwent surgery when its tumor was 310 mm^3^. Mouse 9 **(J)** one day and **(K)** 11 days post-surgery. Secondary tumor growth on the neck of mouse 9 after euthanasia on day 13 post-surgery. Results were obtained from a single experiment.

To observe possible development of PNI/PNS through live imaging, A431 cells expressing luciferase (Luc2) were used. The bioluminescence ability of A431-Luc2 cells were tested prior to tumor challenge and was proportional to the cell number (**Figures 4A, B**). All 10 NSG-A2 mice developed tumors in their whisker pad. Tumors were visible around day 14 and exhibited rapid growth (**Figure 4C**). Tumor volumes on specific surgery days can be visualized on **Table 1**. Local recurrence and metastasis to the neck region were observed respectively in 4/7 (57.1%) and 2/7 (28.6% - M8 and M9) mice that were kept until the experimental endpoint (**Figures 4D, K**).

**Table 1:** Tumor volume on the surgery day and live imaging on the first week post-surgery and endpoint.

Mouse numbers	Surgery day (Days post-tumor challenge)	Tumor volume (mm^3^)	Live Imaging (Days post-surgery)	Endpoint	
				Days post-surgery	Days post-tumor challenge
1	20	490	-	0*	20
2	21	430	-	1*	22
3	25	280	1	1*	26
4	25	220	3	9	34
5	24	260	4	10	34
6	24	320	4	4	34
7	22	350	4	12	34
8	21	490	5	13	34
9	21	310	5	13	34
10	22	360	6	12	34

*Mice that did not survive until experimental endpoint.

### 3.7 Primary tumor resection in the whisker pads reduced tumor burden without compromising the animals’ health and eating habits

Primary tumor resection may be performed in this murine model in order to: 1) Alleviate tumor burden and extend experimental timeline; 2) Potentially improve detection of PNS through imaging methods by decreasing the signal derived from primary tumors on the whisker pad of mice, which could hinder the visualization of PNI/PNS; 3) Reflect the human clinical scenario partially where primary tumors are usually removed months to years prior to PNI/PNS diagnosis.

All 10 NSG-A2 mice underwent primary tumor resection; however, only 7 mice were kept until the experimental endpoint due to complications during the experiment. Three incidents occurred: M1 was culled after surgery due to imperfect surgical technique and wound closing. M2 was found dead on the day 1 post-surgery possibly due to hemorrhage during the night. After this incident, a cautery pen was used instead of cotton tips to stop small bleeding during the surgery. M3 underwent surgery successfully. However, during post-operative assessment the following day, the animal was found with an open wound. This animal was placed under anesthesia and the wound was reglued; however, an excess amount of glue caused the eye and mouth of the animal to be glued shut. Although the excess glue was carefully extracted from the eyes and mouth, the viability of the eye could have been compromised. Therefore, shortly after the incident the mouse was kept under anesthesia to be imaged and immediately euthanized.

A431-Luc2 tumor-bearing NSG-A2 mice did not show any notable weight loss prior to primary tumor resection (**Figure 4E**), except mouse 8 (M8) that showed weight loss of 6.8% from day 2 to day 1 pre-operative followed by 2.2% weight loss on the surgery day (**Figure 4F**). However, these values were below the ethical endpoint score (weight loss > 15%) and indicated moderate changes. On the surgery day, M8 presented with a 490 mm^3^ tumor, suggesting that tumors larger than 500 mm^3^ may compromise the eating habits of animals. In contrast, M1 also had a 490 mm^3^ tumor, but this animal showed normal weight loss (< 5%) (**Figure 4F**).

Performing the resection when tumors were below 500 mm^3^ as well as applying Bepanthen cream on the tumor site helped prevent the occurrence of early ulceration and preserve integrity of the skin flap during the blunt dissection (**Figure 4H**). However, redness on the tumor site on the surgery day was still observed in some mice (**Figure 4I**). In general, surgeries were successful with no signs of infection. Suturing in addition to application of topical skin adhesive proved to be a superior wound closure system than using the topical skin adhesive alone. The skin on the whisker pad of animals appeared mostly recovered by the second week post-surgery (**Figures 4J, K**). On the first day post-operative, animals showed weight loss of 10-15% (**Figure 4G**). However, their weight returned to normal on the second- and third-days post-surgery, suggesting that their eating habits had not been critically affected by the surgery (**Figure 4J**). Animals were able to recover well; general health and behavior were not severely impacted by this procedure.

### 3.8 Bioluminescence patterns suggestive of PNI/PNS did not correlate with histological findings

**Figure 5:**
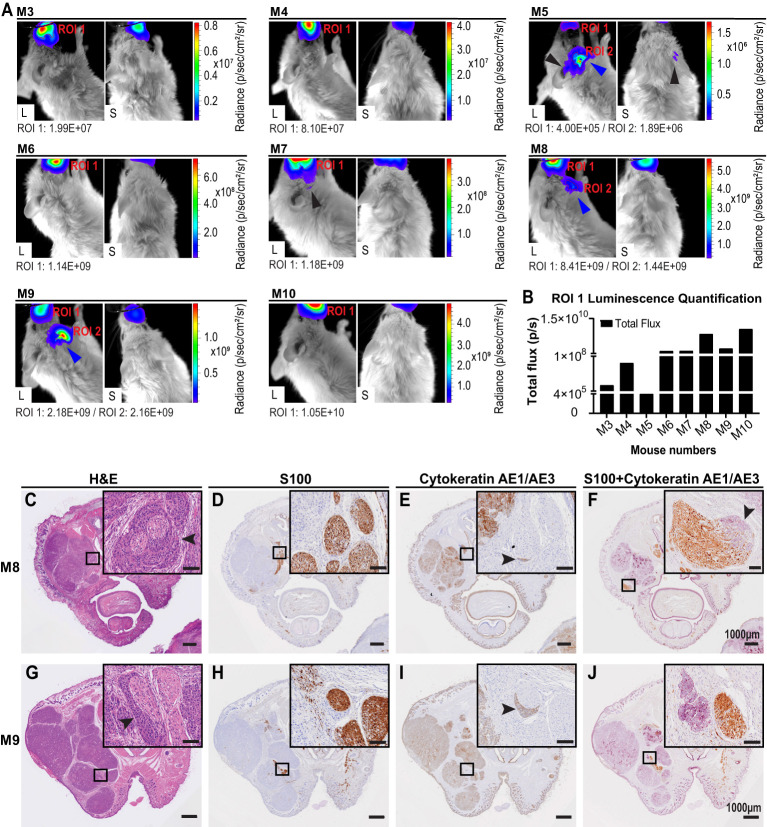
Live imaging and histological analysis of PNI of cutaneous SCCHN in NSG-A2 mice. **(A)** Live imaging of NSG-A2 mice on the first week post-primary tumor resection. Mice (n=8 (7 mice through experimental timeline, one imaged post-surgery but pre-euthanasia as noted (see **Table 1**)) were injected with 15 mg/ml luciferin (IP), anesthetized with 2.5% isoflurane, and imaged. Lateral and superior images were taken at the highest magnification (Field of View: 4 cm). Images show the highest luminescence level representing luciferase saturation. Luminescence was measured using the IVIS imaging system, Living Image^®^ software v4.5.5. Radiance (p/sec/cm^2^/sr) represented by the colour scale is different for each mouse. Black arrows indicate a bioluminescence pattern suggestive of PNI that did not correlate with histology. Blue arrows indicated the bioluminescence patterns suggestive of secondary tumor growth on the neck region. **(B)** Region of Interest (ROI) measured by total flux (photons/second). Graphs were plotted using GraphPad Prism v8.3.1. After euthanasia, mouse heads were removed, fixed in formalin, and decalcified. Samples (n=7) were processed into paraffin wax. Three transverse sections (3-4 μm) were performed at 90°C at three different distances from the primary tumor site. Images show the second section displaying the nasal and oral cavity of animals. **(C, G)** H&E stain. **(D, H)** IHC stain for S100 (nerves). Sections were incubated with anti-S100 and secondary MACH1 Universal HRP anti-rabbit. **(E, I)** IHC stain for cytokeratin (cutaneous SCC). Incubation with anti-pan cytokeratin (AE1/AE3) was followed by secondary MACH2 HRP 221 anti-mouse. **(F, J)** Dual IHC for S100 and cytokeratin. S100 was stained with DAB (brown) and pan cytokeratin with Vector purple (purple). Arrowheads indicate intraneural and perineural invasion observed in mouse 8, and PNI in mouse 9. Data shown are the results of a single experiment. Slides were scanned at 40× magnification using the VS120 Olympus Slidescanner. Scale bars: 1000 µm and 100 µm (insets). Data were obtained from a single experiment. L, Lateral; S, Superior.

Eight NSG-A2 mice were imaged on different post-surgery days that are specified on the **Table 1**. Bioluminescence was observed on the primary tumor site of all mice, including M3 which was euthanized on day 1 post-surgery (**Figure 5A**), indicated the presence of remaining A431-Luc2 cells after excision (**Figures 5A, B**). This suggested that the primary tumor resection was not 100% complete, which was expected considering that we did not have the necessary margins to clear the tumor (at least a 5 mm) in this model. Bioluminescence pattern observed on M5 (lateral and superior view) and M7 (lateral view) (**Figure 5A**), indicated by black arrows, seemed to suggest PNI/PNS; however, these patterns did not correlate with the histological analysis (**Figures 5C–J**). Therefore, bioluminescence imaging was not sufficient to observe PNI/PNS *in vivo*. Bright signal on the neck region indicated metastasis on mice 5, 8, and 9 (blue arrows, **Figure 5A**). Secondary tumor growth on the neck of mice 8 and 9 was visible on the second week post-surgery (endpoint), but not on M5. Quantification of luminescence from the primary sites are observed on **Figure 5B**. Animals were euthanized on day 34 post-tumor challenge and on different post-surgery days (**Table 1**).

Analysis of stained slides by the independent pathologist detected PNI in 2/7 (28.6%) animals that were kept until the endpoint, however no PNS was detected. Metastasis to the neck region was concomitant with PNI on the same animals. PNI was only detected on the second transverse section (**Figure 1**) exhibiting the nasal and oral cavity of animals (**Figures 5C–J**). Intraneural and perineural invasion was observed on M8 in different nerves (**Figures 5C–F**). The H&E stain displayed characteristic onion skin-like pattern (**Figure 5C**), as described by (3). Intraneural invasion was only clearly observed on the dual IHC staining (**Figure 5F**). PNI was also visualized in M9 in both H&E and separate IHC staining (**Figures 5G–I**); however, it could not be observed on the dual IHC analysis (**Figure 5J**). Moreover, tumor areas and margins can be visualized in the transverse sections (**Figures 5C, G**). Malignant cells generally display an epithelioid morphology, nuclear hyperchromasia, and variable nuclear size and shape (3). Size of primary tumors could be correlated with local recurrence of tumor on different post-surgery days.

### 3.9 Decreased perineural invasion following LOXL2 inducible knockdown using the whisker pad model

**Figure 6:**
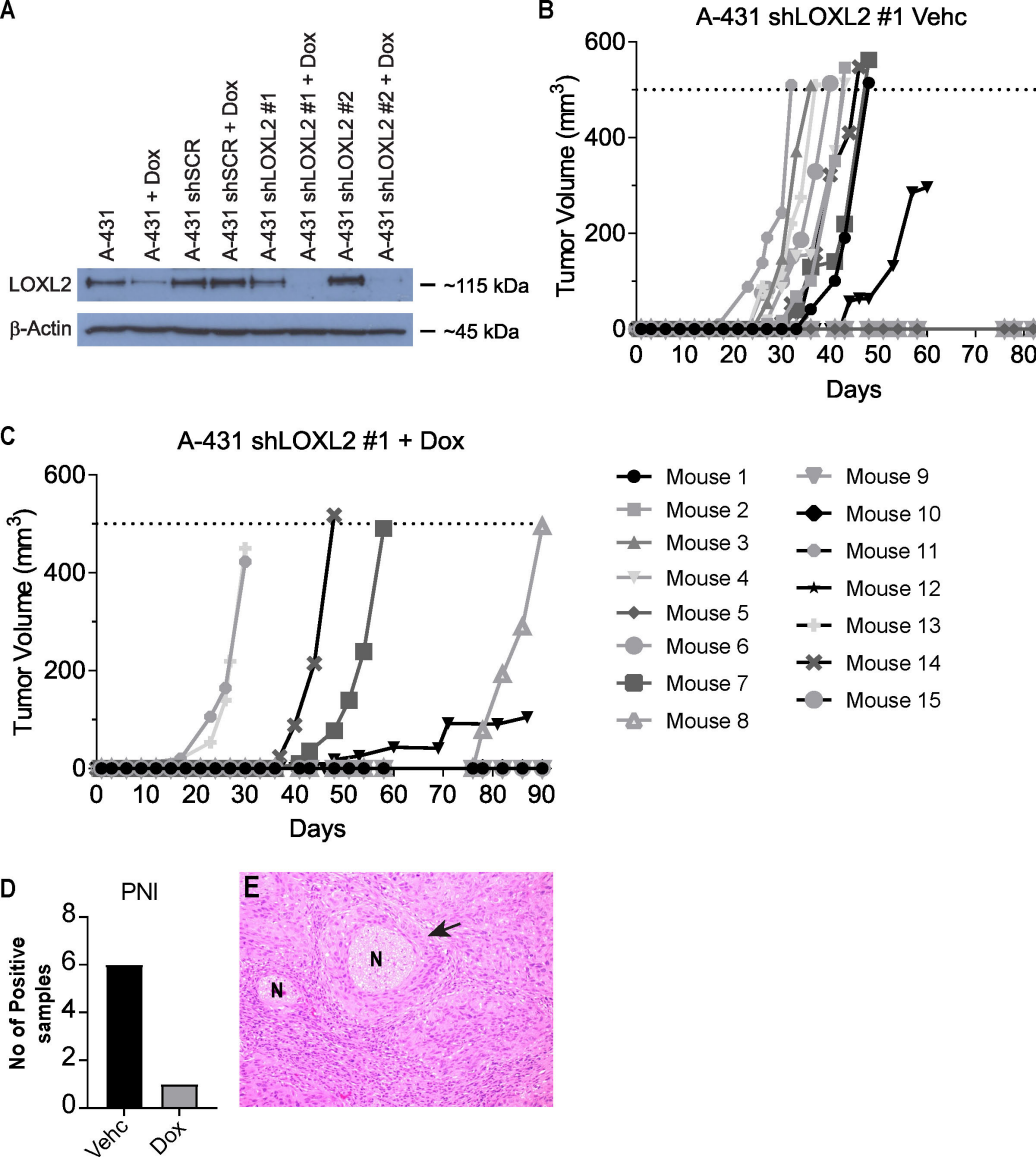
Primary tumor growth of LOXL2 ablated A431-Luc2 cells. **(A)** LOXL2 protein expression in human cutaneous SCC cell line (A431-Luc2) transduced with shRNAs targeting LOXL2 analyzed by Western blotting (2 experimental repeats). Transduced cells were treated with 50 ng/ml doxycycline for 72 h prior to protein harvest. 30 μg protein lysate was loaded and probed with anti-LOXL2 antibody. A431-Luc2 shLOXL2 #1 showed complete silencing of LOXL2 gene expression, while A431-Luc2 LOXL2 #2 showed partial ablation of gene expression. Primary tumor growth in Balb/*c Foxn1^nu^* mice implanted with 1 × 10^3^ A431-Luc2 cells transduced with lentivirus containing shLOXL2 #1 treated with **(B)** 4% sucrose (Vehicle) and **(C)** 2 mg/ml doxycycline in 4% sucrose (n=15 mice per group). **(D)** PNI development in A431-Luc2 shLOXL2 Vehicle and A431-Luc2 shLOXL2 Doxycycline groups. **(E)** Representative H&E image showing PNI detected in a mouse from the A431 shLOXL2 Vehicle (Control) group. Murine data were obtained from 2 experimental repeats. Vehc, Vehicle; Dox, Doxycycline; N, Nerve.

Our previous work has identified the first molecular differences of cSCCHN with PNI compared to cSCCHN without any invasion (32). We identified higher expression of LOXL2 in tumors exhibiting PNS. LOXL2 is a member of the lysyl oxidase (LOX) family that catalyzes the cross-linking of collagens or elastins in the extracellular matrix. Recent studies have also implicated LOXL2 in the regulation of epithelial-to-mesenchymal transition (EMT) and tumor progression. LOXL2 is known to play an important role in regulation of several genes in the EMT cascade. We therefore wished to examine the effects of inducible ablation of LOXL2. We developed an inducible knockdown system to repress expression of LOXL2 using shRNA (**Figure 6A**). We found no phenotype upon inducible silencing of LOXL2 in SCC cell lines *in vitro* (proliferation, growth in agar, invasion; results not shown).

We examined the effect of inducible knockdown of LOXL2 in A431-Luc2 cells injected into the whisker pad of BALB/c *Foxn1* ^nu^ mice. Inducible ablation of LOXL2 expression using lentiviral delivered shRNA reduced incidence of primary tumor growth in the mouse model (LOXL2 ablation with doxycycline addition - 40% (6/15 mice) versus vehicle only - 66% (10/15 mice) (**Figures 6B, C**). Where there was tumor growth, ablation of LOXL2 resulted in 16% PNI development (1/6 mice) in contrast to uninduced control tumors showing 50% PNI (5/10 mice) (**Figures 6D, E**).

## 4 Discussion

Although *in vitro* models replicate some aspects of PNI, they fail to effectively reconstruct the interaction between nerve, tumor, stroma and the immune system (33). *In vivo* models represent the human counterparts more closely and offer reasonable methods of modeling the disease. Several *in vivo* animal models have been described studying cancers that are known to metastasize via the perineural route (44, 45), including oral head and neck SCCs (46).

Previous studies have utilized the sciatic nerve model described by Deborde et al. (38) to study PNI (38, 47–49). We used the sciatic nerve model to observe the extent of cSCC (A431-Luc2 cells) invasion into the nerve. These cells were used as they were most able to induce PNI/PNS. Other cell lines, including UV induced cSCC failed to form PNI/PNS. Our analysis of human PNI/PNS samples (manuscript in preparation) suggest that EGFR expression is important in cSCC nerve invasion, and this is reflected in the cell lines able to form PNI/PNS in our models. We were able to monitor tumor growth through live imaging (**Figure 2**) and confirm neural spread along the nerve with no evidence of tumor outside the nerve by histology (Day 7 and 12). This histological observation fits very well with what we find clinically. Although this model ensures 100% nerve invasion, allows imaging and is an excellent model to test therapies (50), it does not replicate the biological mechanisms of the human disease. The direct injection of cells into the sciatic nerve bypasses crucial steps in the metastatic progression of PNI, including the invasion of the tumor into the membranous layers that construct the nerve. Therefore, it does not allow investigation of disease pathogenesis.

Considering that, we developed a model that more closely mimicked the natural processes of PNI/PNS of cSCCHN. Our model not only allows the investigation of the biological processes involved, but also the modulation of molecular drivers of the process to obtain potentially actionable pathways to prevent this form of metastasis. Moreover, it allows testing of therapies. The two models used in concert, the sciatic nerve model for initial therapeutic testing due to 100% penetrance, followed by best therapeutic strategy testing in the whisker pad model, would be ideal.

Our study is the first to describe an animal model of PNI/PNS of cSCCHN. The aim of our model was to establish primary tumors in a clinically relevant area, with minimal disruption to the local tumor and nerve environment. For an *in vivo* model to be effective in replicating the disease process, few manipulations to the natural environment should be made. We used the whisker pad of mice, as it is a facial area richly innervated by sensory nerve endings from the maxillary division of the trigeminal nerve (V_2_). However, the V_1_ ophthalmic branch could be potentially used instead to reduce any distress caused by tumor growth and surgical resection on the whisker pad.

Using this region of the whisker pad allows us to effectively model the human disease counterpart in three ways. Firstly, the trigeminal nerve is one of the most frequently involved cranial nerves, specifically the V_2_ branch, as observed in clinic (29). Secondly, our model allows the tumor and metastasis to form in a tissue relevant context. Squamous cells are found within the epidermis, the most superficial layer of the skin. However, injecting a cell suspension into the epidermis is difficult to achieve due to the thinness of this layer. Injecting into the subcutaneous space offers an appropriate and technically achievable alternative. Lastly, this model allows the natural progression of the disease, without extensive biological and structural alterations to the local tissue. Establishing the tumor in the subcutaneous space requires the tumor to invade through several layers of underlying tissue, meaning that only tumors with the propensity to invade a nerve will do so. In our study, we found that the optimal starting cell number for tumor growth was 2.0 × 10^4^ cells/site. From this cohort, 1/10 (10%) and 2/7 (28.6%) tumors developed PNI/PNS in BALB/c *Foxn1* ^nu^ and NSG-A2 mice, respectively. Despite this low rate, this remains an important discovery as it shows that not all tumors established in this area will invade into the perineural space and it is comparable to the clinical scenario (2-14% PNI incidence in cSCCHN). Importantly, it also shows that this type of invasion is possible in this region of a mouse. We hypothesize that the rate of PNI development in this model might be increased by only resecting tumors when they reach approximately 500 mm^3^. Recurrent and large as well as poorly to moderately differentiated tumors have been shown to be more likely associated with PNI in several studies (9, 15, 25, 31, 51–53). Moreover, our results showed that the detection of PNI was associated with recurrence and metastasis to the neck region of NSG-A2 mice, while no PNS was observed in this cohort. This finding corroborates with the clinical scenario where studies have shown the correlation between PNI and high local recurrence and lymph node metastasis after surgery/RT (19–22). Although PNS carries a worse prognosis than incidental PNI, it has been shown that nodal metastasis in PNS is not as common as in incidental PNI, suggesting that perineural tumor spread occurs differently from metastasis via a lymphatic or hematogenous route (54–57).

The molecular mechanisms of PNI are poorly understood, though several molecular alterations in genes such as *LOXL2*, *TGM3*, *TP53* and *FGFR2* have been implicated in this process (32, 58). The application of our model to molecular studies would allow for an *in vivo* functional validation of mutations or protein alterations that may be associated with PNI/PNS. The low rate of PNI, and importantly, the possibility of PNS using our model, shows its applicability in the study of gene modifications hypothesized to be involved in driving PNI/PNS. The utility of this model is demonstrated with the inducible knockdown of LOXL2 inhibiting primary tumor growth as well as PNI. The percentage of PNI development in the uninduced control (vehicle) group was higher (50% - 5/10 mice where the tumors grew) when compared to the doxycycline-induced shLOXL2 cohort (about 16% PNI - 1/6 mice). This result suggests a role for LOXL2 in PNI development and the usefulness and validity of this model for investigating potential PNI targets. The vehicle control group (A431-Luc2 -shLOXL2 cells) showed a higher level of PNI development when compared to the A431-Luc2 experiment (50% PNI (5/10 mice), **Figure 6D**, 1.0 × 10^3^ cells/site vs 28.6% PNI (2/7 mice), **Figures 5C–J**, 2.0 × 10^3^ cells/site). As the A431-Luc2-LOXL2 cells went through two additional rounds of selection with different viruses (transduction with pLENTI6-TR, then pLENTI4/TO/shLOXL2), compared to the A431-Luc2 cells, the variation in the results could be explained by possible alteration in phenotype of A431-LOXL2 resulting cells which enabled more frequent PNI. Polyclonal selection was used following virus transduction, making these differences unexpected. Future experiments should consider multifunctional lentiviral constructs with all desired elements in a single vector.

In recent years, unbiased short-hairpin RNA and CRISPR (clustered regularly interspaced short palindromic repeats) based gene-targeting libraries have been used to identify genes involved in different cellular processes, including metastasis (59). To identify drivers of PNI in cSCCHN, similar high-throughput screens could be prepared in other keratinocyte cell lines, and then applied *in vivo* using methods outlined in this manuscript. If a gene modification is associated with a more aggressive tumor type with the propensity to invade the perineural space, one would expect the expression of such a molecular aberration would alter the rate of PNI in our model. Our model portrays the human counterpart of cSCCHN in such a way that the tumor grows and invades in a similar environment. The tumor must invade multiple layers of tissue, so PNI will not occur unless the tumor has a molecular profile for PNI.

We further demonstrated that this method can be modified to become a longer-term study of PNI/PNS. We showed that primary tumor resection in mice was feasible, enabled us to extend the length of the experimental timeline and fit with the human clinical scenario to a certain extent where primary tumors are removed months to years prior to PNS. Additionally, we used bioluminescence imaging, as it is useful to monitor therapy response in preclinical models (60, 61). However, bioluminescence results did not correlate with histological findings in our whisker pad model. Therefore, magnetic resonance imaging may offer a better resolution image to facilitate visualization of PNI in our model and could be investigated further. Moreover, we used two different strains of mice in this study, BALB/c *Foxn1* ^nu^ and NSG-A2. To better reflect the human disease, the immunocompetent NSG-A2 mice can be further humanized to test potential targeted therapies, immunotherapies and to investigate the interaction between human cancer and immune cells in nerves.

A limitation of our model is that our optimization was performed on a single cell line. For future experiments using other keratinocyte-lineage cell lines, the starting cell densities should be optimized to achieve moderate rate of tumor growth, reliable tumor formation. and to uncover the baseline PNI rates for individual cell lines, considering that other cell lines may yield higher baseline rates of PNI. Another limitation of our method is that an advanced level of technical skill is required to perform consistent subcutaneous injection and primary tumor resection. The risk of introducing cells directly into nerve and nasal or oral cavities increases with poor injection technique. The appearance of translucent bleb in the skin after injection of tumor cells should be observed to ensure correct injection depth. If this is not achieved, it should be noted and considered when assessing rates of PNI.

There are some important considerations to this method. Delivery of the cell suspension with a 31G or finer needle minimally disrupts the structural anatomy of the area, minimizing damage to the local tissue and unnecessary inflammation. Further, a single unilateral injection allows for a longer monitoring period, as tumors are able to grow larger. This reduces the possibility of early experimental termination due to respiratory abnormalities or feeding behaviors (i.e. blockage to the nasal cavity or obstruction of the oral cavity due to tumor growth).

To the best of our knowledge, there are currently no other available *in vivo* models of PNI/PNS in cSCCHN that model the disease processes to this extent. We anticipate in developing this model that it will contribute to the understanding of the molecular mechanisms of the process and allow testing of potential therapies. A detailed understanding of the mechanisms underlying PNI and PNS is of paramount importance before effective therapies can be designed to prevent or treat this type of invasion.

## Data availability statement

The original contributions presented in the study are included in the article/**Supplementary Material**. Further inquiries can be directed to the corresponding author.

## Ethics statement

The animal study was reviewed and approved by QIMR Berghofer Animal Ethics Committee and the University of Queensland Animal Ethics Committee.

## Author contributions

PL and NB performed, analyzed murine experiments, composed figures drafted manuscript sections and assembled data/figures. PL performed and analyzed experiments with NSG-A2 mice. NB performed and analyzed experiments evaluating the LOXL2 inducible knockdown using Balb/c nude mice, immunohistochemistry and Western blotting. JH helped with the whisker pad model using NSG-A2 mice and performed primary tumor resections. JL and DG performed optimization experiments to establish the whisker pad model. IB. as an accredited Pathologist examined stained slides to confirm the presence of PNI/PNS. BP provided guidance on the clinical situation and need relating to cSCCHN PNI, helped develop the whisker pad model and co-supervized students. GB conceptualized and developed the whisker pad model, authored parts of this manuscript, made the lentiviral constructs described in the manuscript, transduced and selected the A431 cells, and supervised students. FS conceptualized the development of the whisker pad model to use of intravital imaging for therapy testing, authored and edited manuscript, designed experiments, analyzed data, was responsible for ethics, governance and research integrity checks and was principal supervisor for PL. 
