# Supplementary material for: Development of an in vivo murine model of perineural invasion and spread of cutaneous squamous cell carcinoma of the head and neck
Source: Front Oncol. 2023 Sep 7;13:1231104. doi: 10.3389/fonc.2023.1231104 (PMC10513369; doi:10.3389/fonc.2023.1231104)
Supplement: Supplementary file 1 [file DataSheet_1.pdf]

Supplementary Material

Development of an *in vivo* murine model of perineural invasion and spread of cutaneous squamous cell carcinoma of the head and neck

Priscila O. de Lima[1]†, Natasa Broit[2]†, Johnson D. Huang[1], Jae H. Lim[2,4,6], Damien J. Gardiner[2,4], Ian S. Brown[3,5], Benedict J. Panizza[3,4]†, Glen M. Boyle[2,3,7]† and Fiona Simpson[1]†*.

*** Correspondence:** Fiona Simpson. Email: f.simpson@uq.edu.au

Table 1: Key reagents and resources.

**Reagent type or resource**		**Source**	**Identifiers**	**Additional information**
Organisms/ Strains	BALB/c *Foxn1*^{nu} mice (*A/A* $Tyrp1^{b}/Tyrp1^{b}$ Tyr^{c}/Tyr^{c}, MHC haplotype ($H2K^{d}$) and C5 normal	Animal Resource Centre	Product code: BCNU; RRID:MGI:5652590	Sex: Males.
	NSG-HLA-A2.1 mice (NOD.Cg-$Mcph1^{Tg(HLA-A2.1)1Enge}$ $Prkdc^{scid}$ $Il2rg^{tm1Wjl}$/SzJ)	Radford laboratory (Mater Medical Research) originally sourced from the Jackson Laboratory	Stock#009617; RRID:IMSR_JAX:009617	Sex: Males and females were used.
Cell lines	A431	ATCC	Cat#CRL-1555; RRID:CVCL_0037	
	A431-Luc2 and A431-Luc2-shLOXL2	Prof. Glen Boyle (Simmons, Pierce, Al-Ejeh, & Boyle, 2017) DOI:10.1038/s41598-017-11366-y		

Antibodies	Rabbit monoclonal anti-human LOXL2 (EPR12733)	Abcam	Cat#ab179810	WB (1:1000)
	Rabbit monoclonal anti-β-actin	Cell Signaling Technologies (CST)	Cat#4970; RRID:AB_2223172	WB (1:2000)
	Mouse anti-human pan cytokeratin (clone AE1/AE3)	Agilent DAKO	Cat#M3515/M351529; RRID:AB_2132885	IHC (1:300)<br>Dual IHC (1:250)
	Rabbit polyclonal anti-S100	Leica Biosystems	Cat#NCL-L-S100p; RRID:AB_564003	IHC (1:160)<br>Dual IHC (1:250)
	Goat polyclonal anti-rabbit IgG	Cell Signaling Technologies (CST)	Cat#7074; RRID:AB_2099233	No dilution required. Ready to use
	MACH2 goat anti-rabbit HRP	Biocare Medical	Cat#RHP520	No dilution required. Ready to use
	MACH2 goat anti-mouse HRP	Biocare Medical	Cat#MHRP520	No dilution required. Ready to use
Recombinant DNA	pLENTI6/TR	Invitrogen	Cat#K4965-00	
	pLENTI4/TO/V5-DEST	Invitrogen	Cat#K4965-00	
	shLOXL2 sequences cloned into pcDNA6.2-GW-EmGFP	Invitrogen.	Hmi409846_top_LOXL2; Hmi409846_bot_LOXL2; Hmi409847_top_LOXL2;	Transduced into A431cells.

			HMI409847_bot_LOXL2	
	pLENTI6/luciferase	Glen Boyle (Simmons, Pierce, Al-Ejeh, & Boyle, 2017)<br><br>DOI:10.1038/s41598-017-11366-y		A431 cells were transduced with lentivirus containing pLENTI6/luciferase.
Commercial assay or kit	MACH1 Universal anti-rabbit HRP-Polymer Detection	Biocare Medical	Cat#M1U539	
Chemical compound or drugs	Ketamine	Clifford Hallam Healthcare	Cat#1880206;<br><br>CAS ID: 6740-88-1	Administered dose: 100 mg/kg.
	Xylazil-20 (Xylazine)	Clifford Hallam Healthcare	Cat#1945536;<br><br>CAS ID: 7361-61-7	Administered dose: 10 mg/kg.
	Buprenorphine	Clifford Hallam Healthcare	Cat#1238366; CAS ID: 52485-79-7	Administered dose: 0.05 mg/kg
	Carprofen (Rimadyl)	Zoetis	Cat#10001132; CAS ID: 53716-49-7	Administered dose: 5 mg/kg
	D-luciferin potassium salt	Gold Biotechnology	Cat#LUCK; CAS ID: 115144-35-9	Stock: 15 mg/mL. Final concentration / well: 150 µg/ml.
	10% Neutral buffered formalin	Australian Biostain	Cat#ANBFB.5; CAS ID: 50-00-0	

	Vector ImmPACT DAB HRP chromogen (Brown)	Abacus ALS	Cat#VESK4105
	Vector ImmPACT DAB HRP chromogen (Purple)	Abacus ALS	Cat#VESK4605
	Peroxidazed 1	Biocare Medical	Cat#PX968M
	Background Sniper	Biocare Medical	Cat#BS966
	Da Vinci Green antibody diluent	Biocare Medical	Cat#PD900
	Mouse on Mouse (M.O.M.) Blocking Reagent-Rodent Block M	Biocare Medical	Cat#RBM961L
Software and algorithms	ImageJ	[40] DOI:10.1038/nmeth.2089	https://imagej.nih.gov/ij/; RRID:SCR_003070
	Fiji (Image J)	[41] DOI:10.1038/nmeth.2019	https://fiji.sc/ RRID:SCR_002285
	PRISM V7 and V8	GraphPad software	https://www.graphpad.com; RRID:SCR_002798
	Aperio ImageScope	Leica Biosystems	https://www.leicabiosystems.com/digital-pathology/mana

			ge/aperio-imagescope/<br><br>RRID:SCR_020993
	OlyVia	Olympus Life Sciences	https://www.olympus-lifescience.com/en/support/downloads/#!dlOpen=%23detail847249644<br><br>RRID:SCR_016167
	Living Image	Perkin Elmer	https://www.perkinelmer.com/uk/Product/li-software-for-lumina-1-seat-add-on-128110<br><br>RRID:SCR_014247
Other	DAPI	Sigma-Aldrich	Cat#D9542-10MG; CAS ID: 28718-90-3
	DMEM-F12	Life Technologies, Gibco	Cat#11320033
	RPMI-1640	Life Technologies, Gibco	Cat#31800089
	Dulbecco’s PBS	Life Technologies, Gibco	Cat#14190-144
	HEPES	Life Technologies, Gibco	Cat#15630080
	L-Glutamine	Life Technologies, Gibco	Cat#25030081

	Penicillin-Streptomycin	Life Technologies, Gibco	Cat#15140122; CAS ID: 3810-74-0 and 69-57-8	
	Cautery pen Gemini, tip length 5 mm	Able Scientific	Cat#ASCCK-1700/ ASCC-1065	
	Dermabond Advanced Topical Skin Adhesive	Ethicon, Johnson & Johnson	Product Code: ANX12	
	Povidone-Iodine Prep pads	Medical Industries	Cat#LML-08	
	Bepanthen® antiseptic cream	Bayer Pharmaceutics	AUST-R 182923	
	DietGel boost	Clear H_2O	Cat#72-04-5022	
	10 µL Hamilton syringe (model 701RN)	Hamilton	Cat#7635-01	
	30 Gauge, small hub RN needle	Hamilton	Cat#HAMC7803-07	Needle length: 10mm, point style: 4, bevel: 45
	Vannas-type micro scissors, straight 80-82mm	ProSciTech	Cat#TY-3001 or #PEL1346	
	Pointed tweezers, style 5.	ProSciTech	Cat#T65-SS	
	Curved Dumont Dumostar tweezer, style 7	TED PELLA, Proscitech	Cat#527	
	6-0 Vicryl rapide suture	Ethicon	Cat#W9913	

Tissue Plus O.C.T. Compound	Scigen	Cat#4586
Disposable base mold 37x24x5mm	VWR	Cat#SIMPM475 -5
